# Draft Genome Sequence of “Candidatus Izimaplasma sp.” Strain ZiA1, Obtained from a Toluene-Degrading and Iron-Reducing Enrichment Culture

**DOI:** 10.1128/MRA.00861-18

**Published:** 2018-08-30

**Authors:** So-Jeong Kim, Soo-Je Park, Jong-Geol Kim, Man-Young Jung, Joo-Han Gwak, Sung-Keun Rhee

**Affiliations:** a Geologic Environment Research Division, Korea Institute of Geoscience and Mineral Resources, Daejeon, Republic of Korea; b Department of Biology, Jeju National University, Jeju, Republic of Korea; c Department of Microbiology, Chungbuk National University, Cheongju, Republic of Korea; d Division of Microbial Ecology, Department of Microbiology and Ecosystem Science, University of Vienna, Vienna, Austria; Queens College

## Abstract

Here, we report the draft genome sequence of “Candidatus Izimaplasma sp.” strain ZiA1 (1.88 Mb and 29.6% G+C content). Strain ZiA1 was cocultured with iron-reducing and toluene-degrading bacteria in an enrichment culture from tidal flat sediment. Like the genomes of other strains of “*Ca*. Izimaplasma,” the ZiA1 genome contained genes required for anaerobic fermentation.

## Abstract (precis)

Here, we report the draft genome sequence of “Candidatus Izimaplasma sp.” strain ZiA1 (1.88 Mb and 29.6% G+C content).

## ANNOUNCEMENT

The phylum Tenericutes harbors “Candidatus Izimaplasma” as well as the class Mollicutes and unclassified members. Members of “Ca. Izimaplasma” were first reported in 2016 (1). To date, only two bacterial genomes (HR1 and HR2) from an enrichment culture of “Ca. Izimaplasma” have been identified from methane seeps (1). In a previous study, a metagenome-assembled genome related to Tenericutes was obtained from a toluene-degrading and iron-reducing enrichment culture from tidal flat sediment (2). In the current study, we further characterized the genome obtained from the enrichment. On the basis of the results of genomic studies, this genome represents the third genome of “Ca. Izimaplasma.”

The metagenome DNA libraries were prepared from heavy fraction DNA using a TruSeq DNA library. The sequence reads were obtained with a HiSeq 2000 instrument using a paired-end sequencing method (101 × 2 bp). The Sickle tool was used for quality filtering of the raw reads. The filtered reads were assembled using IDBA_UD (ver 1.1.1). Bins were obtained based on coverage and tetranucleotide frequency (3). Automatic annotation was performed using PGAP (ver. 4.2) (NCBI). Finally, based on the taxonomic analysis of the assembled genome bins, one genome bin (34 scaffolds, 1.88 Mb in length) related to “Ca. Izimaplasma” was isolated (Table 1). The draft genome sequence was named ZiA1. Based on CheckM (4), the genome is estimated to be 98.7% complete, with an average G+C content of 29.6%. Further, 1,803 coding sequences (CDS) and 34 tRNAs were identified in the genome. Unfortunately, no rRNA gene sequences were contained in this bin, and therefore all phylogenetic information was predicted using the *rpoB* gene. RpoB of ZiA1 showed 81% amino acid identity against that of HR1 and HR2. Average nucleotide identity (ANI) analysis results between ZiA1 and HR1 and HR2 using JSpeciesWS (5) are presented in Table 1. Furthermore, average amino acid identity (AAI) analysis using CompareM (https://github.com/dparks1134/CompareM) showed that AAIs of strain ZiA1 to HR1 and HR2 were 62.6% and 63.1%, respectively. According to Konstantinidis et al. (6, 7), this result indicates that strain ZiA1 is a novel species in this genus.

**TABLE 1:** Genomic features of “*Candidatus* Izimaplasma sp.” strains ZiA1, HR1, and HR2

Feature	ZiA1	HR1^*a*^	HR2^*a*^
Size (Mbp)	1.88	1.88	2.12
G+C content (%)	29.6	31.3	29.2
No. of scaffolds	22	1	78
No. of genes	1,877	1,846	2,284
No. of rRNAs	0	4	3
No. of tRNAs	34	38	58
No. of protein-coding genes	1,803	1,794	2,222
Completeness (%)	98.7	100	92.1
Contamination (%)	1.3	1.3	1.3
GenBank accession no.	NQYJ00000000	CP009415	JRFF00000000
ANI (%)			
ZiA1		69.98	70.50
HR1	69.80		71.09
HR2	70.48	71.17	

a Data from Skennerton et al. (1).

The ZiA1 genome contained no genes related to toluene degradation but did contain a complete gene set for glycolysis and pyruvate-to-lactate conversion. Most genes required for the tricarboxylic acid (TCA) cycle were missing, indicating that these bacteria use strict anaerobic fermentation metabolism for substrate-level phosphorylation. Based on the genomic analysis, strains HR1 and HR2 can utilize glucose, sucrose, and maltose for fermentation. The genome of ZiA1 contained genes for glucose fermentation, while genes required for sucrose and maltose fermentation were absent. Similar to strains HR1 and HR2, hydrogenases coupled with ferredoxin and NADP, two Rnf complexes, and F_o_F_1_ ATP synthase required for energy metabolism were present. The Na^+^-transporting NADH:ubiquinone oxidoreductase (NqrBCDEF) was observed only in this genome. In the phylum Tenericutes, only Acholeplasma palmae was known to have an Na^+^-transporting NADH:ubiquinone oxidoreductase complex (8). According to Skennerton et al. (1), cytochrome *bd* oxidase of “*Ca*. Izimaplasma” was found to be a genomic feature distinct from the class Mollicutes. However, in the current study, genes for cytochrome *bd* oxidase were not found in the genome of strain ZiA1.

### Data availability.

The accession numbers of the draft genome sequences of “Candidatus Izimaplasma sp.” ZiA1 are given in Table 1.
